# LmbU, a Cluster-Situated Regulator for Lincomycin, Consists of a DNA-Binding Domain, an Auto-Inhibitory Domain, and Forms Homodimer

**DOI:** 10.3389/fmicb.2019.00989

**Published:** 2019-05-03

**Authors:** Bingbing Hou, Xiaoyu Zhu, Yajing Kang, Ruida Wang, Haizhen Wu, Jiang Ye, Huizhan Zhang

**Affiliations:** ^1^State Key Laboratory of Bioreactor Engineering, East China University of Science and Technology, Shanghai, China; ^2^Department of Applied Biology, East China University of Science and Technology, Shanghai, China

**Keywords:** LmbU, CSR, functional domain, HTH, homodimer, regulatory mechanism

## Abstract

Few studies were reported about the regulatory mechanism of lincomycin biosynthesis since it was found in 1962. Although we have proved that a cluster-situated regulator (CSR) LmbU (GenBank Accession No. ABX00623.1) positively modulates lincomycin biosynthesis in *Streptomyces lincolnensis* NRRL 2936, the molecular mechanism of LmbU regulation is still unclear. In this study, we demonstrated that LmbU binds to the target *lmbAp* by a central DNA-binding domain (DBD), which interacts with the binding sites through the helix-turn-helix (HTH) motif. N-terminal of LmbU includes an auto-inhibitory domain (AID), inhibiting the DNA-binding activity of LmbU. Without the AID, LmbU variant can bind to its own promoter. Interestingly, compared to other LmbU homologs, the homologs within the biosynthetic gene clusters (BGCs) of known antibiotics generally contain N-terminal AIDs, which offer them the abilities to play complex regulatory functions. In addition, cysteine 12 (C12) has been proved to be mainly responsible for LmbU homodimer formation *in vitro*. In conclusion, LmbU homologs naturally exist in hundreds of actinomycetes, and belong to a new regulatory family, LmbU family. The present study reveals the DBD, AID and dimerization of LmbU, and sheds new light on the regulatory mechanism of LmbU and its homologs.

## Introduction

*Streptomycetes* are well known as prolific producers of bioactive secondary metabolites including more than half of antibiotics as well as antitumor agents, antifungal compounds and vitamins, which have remarkable pharmacological, and industrial importance. Biosynthetic genes for antibiotics and other secondary metabolites are typically clustered together on the chromosomes (Cundliffe, 2006; Liu et al., 2013) or the plasmids (O’Rourke et al., 2009), designated as BGC, and are subject to multi-level and complex regulation cascades. Among them, CSRs provide direct contributions to the biosynthesis of antibiotics by responding to pleiotropic regulators (Ohnishi et al., 2005), global regulators (Uguru et al., 2005; Higo et al., 2011; Iqbal et al., 2012), and different kinds of regulatory small molecules (Wang et al., 2009; Zhang et al., 2013), subsequently regulating expression of the other biosynthetic genes within their cognate clusters. However, not all the BGCs share a same regulatory scheme. Some of them, such as jadomycin BGC, harbor multiple CSRs (Zou et al., 2014), some of them, such as sansanmycin BGC, contain a single CSR (Li et al., 2013), while the others, such as lincomycin BGC, lack any distinct CSRs (Hou et al., 2017). Generally, CSRs belong to a variety of regulatory protein families, which are divided by sequence or structural similarities, including SARP (*Streptomyces* antibiotic regulatory protein) family, LAL (large ATP-binding regulators of the LuxR) family, TetR family, and so on.

The most common and best studied CSRs are those of the SARP family in *Streptomyces*, including ActII-ORF4 within actinorhodine BGC as well as RedD within undecylprodigiosin BGC in *Streptomyces coelicolor* (Takano et al., 1992; Arias et al., 1999), DnrI within daunorubicin BGC in *Streptomyces peucetius* (Sheldon et al., 2002), and CcaR within cephamycin-clavulanic acid BGC in *Streptomyces clavuligerus* (Santamarta et al., 2011). These members are classified by having an N-terminal HTH DBD subject to OmpR-type and a transcriptional activation domain (Wietzorrek and Bibb, 1997), which generally positively regulate the biosynthesis of secondary metabolites (Sheldon et al., 2002; Tanaka et al., 2007). The target promoters of these members usually contain direct repeats, for instance, direct heptameric repeats (5’-TCGAGXX-3’) with 4 bp spacers are conserved upstream the -10 regions of the promoters targeted by ActII-ORF4 and DnrI (Tanaka et al., 2007). The LAL family members usually function as activators in *Streptomyces* too, and comprise an N-terminal ATP-binding domain with a C-terminal LuxR-type DBD, including PikD within pikromycin BGC in *Streptomyces venezuelae* (Wilson et al., 2001), RapH within rapamycin BGC in *Streptomyces hygroscopicus* (Kuscer et al., 2007), and AveR within avermectin BGC in *Streptomyces avermitilis* (Guo et al., 2010). Compared to the SARP and LAL families, TetR family members are widely distributed in various bacteria, including ActR within actinorhodin BGC in *S. coelicolor* (Tahlan et al., 2008), TetR in *Escherichia coli* (Kisker et al., 1995), RolR in *Corynebacterium glutamicum* (Li et al., 2012), LplR in *Rhodococcus erythropolis* (Si et al., 2012), and VtpR in *Vibrio tubiashii* (Hasegawa and Häse, 2009). The TetR family members usually function as transcriptional repressors and consist of an N-terminal DBD and a C-terminal functional domain, which can bind to one or more ligands, subsequently losing the DNA-binding activity and turning on transcription of the target genes (Yu et al., 2010; Cuthbertson and Nodwell, 2013).

Previously, we have reported that LmbU functions as an activator belonging to a novel regulatory family, LmbU family (Hou et al., 2017; van der Heul et al., 2018), and promotes lincomycin biosynthesis by directly regulating transcription of the biosynthetic genes (Hou et al., 2017). The conserved binding site of LmbU is a palindromic sequence 5’-CGCCGGCG-3’, which was found in the promoter regions of the *lmbA* and *lmbW* genes. While, the regulatory mechanism of LmbU to other genes lack of the conserved motif is still unknown. In addition, because LmbU and its homologs have no significant sequence and structural similarities to other known CSRs, the binding pattern and functional domains of LmbU are also unclear. In the present study, we characterized the DBD (HTH motif) of LmbU and demonstrated that N-terminal of LmbU contains an AID, which was found in LmbU homologs within the BGCs of antibiotics, but not in that outside BGC of antibiotics. LmbU inhibits transcription of its own gene *in vivo*, and LmbU variant without AID can bind to *lmbUp* promoter. In addition, we revealed that LmbU can form homodimer by a disulfide bond *in vitro*.

## Materials and Methods

### Homology Modeling, Sequence Alignment, and Phylogenetic Tree Analysis

Secondary structure analysis of LmbU was performed by using an online software PredictProtein^1^. Homology modeling of LmbU was performed by using an online software SwissModel^2^. The templates used for LmbU modeling were chosen by ranking, including *Tt*Spo0J derived from *Thermus thermophiles* (GenBank Accession No. WP_011173975.1), *Hp*Spo0J derived from *Helicobacter pylori* (GenBank Accession No. ACJ08256.1), AtaR derived from *E. coli* (GenBank accession no. 6AJN_F), ParG derived from a multidrug resistance plasmid TP228 from *E. coli* (GenBank Accession No. ACV89876.1), AmrZ derived from *Pseudomonas aeruginosa* (GenBank Accession No. APJ53923.1), ω repressor derived from a plasmid Psm19035 from *Streptococcus pyogenes* (GenBank accession no. AAR27202.1), and Arc repressor derived from *Salmonella* bacteriophage P22 (GenBank Accession No. AAM81381.1). Sequence alignment of LmbU with its homologs and the targets of LmbU were carried out by using DNAMAN (Hou et al., 2017). Phylogenetic tree analysis was inferred by using MEGA v7.0.14 with the maximum likelihood method, the LmbU homologs were chosen by ranking (Bown et al., 2017; Hou et al., 2018).

### Bacterial Strains, Plasmids, and Growth Conditions

The bacterial strains and plasmids used in this study are listed in Table 1. *E. coli* JM83, BL21 (DE3) and ET12567/pUZ8002 strains were used for plasmids construction, protein overexpression and *E. coli*-*Streptomyces* conjugation, respectively. *Streptomyces lincolnensis* wild-type strain NRRL 2936 and *lmbU* disruption strain JLUa2 were used for *xylTE* reporter assays *in vivo* (Mao et al., 2015; Hou et al., 2018). The pET-28a (+) plasmid was used for overexpression of LmbU and its variants, and the pIB139 plasmid was used for *xylTE* reporter analysis. *E. coli* strains were grown in liquid or on solid Luria-Bertani media at 37°C. *S. lincolnensis* NRRL 2936 and mutants were grown in liquid YEME medium or on solid SMA and ISP4 media at 28°C as described previously (Hou et al., 2017). The media were added with 50 μg/ml kanamycin, 50 μg/ml apramycin, and 30 μg/ml chloramphenicol as appropriate.

**Table 1:** Strains and plasmids used in this study.

Strain or plasmid	Genotype and/or description	Source or reference
**Strains**		
***S. lincolnensis***		
NRRL 2936	Wild-type, lincomycin producer	NRRL, United States
JLUa2	NRRL 2936 Δ*lmbU*	Hou et al., 2017
LNA	NRRL 2936 attBΦC31::pATE152	Hou et al., 2018
LUA	JLUa2 attBΦC31::pATE152	Hou et al., 2018
JAU01	JLUa2 attBΦC31::pAU01	This study
JAU02	JLUa2 attBΦC31::pAU02	This study
JAU03	JLUa2 attBΦC31::pAU03	This study
JAU06	JLUa2 attBΦC31::pAU06	This study
JAU07	JLUa2 attBΦC31::pAU07	This study
LNU	NRRL 2936 attBΦC31::pUTE152	This study
LUU	JLUa2 attBΦC31::pUTE152	This study
***E. coli***		
JM83	F’, ara, Δ(*lac-pro* AB), *rpsL*, (Str^r^), Φ80, *lacZ*ΔM15	Hou et al., 2017
BL21 (DE3)	F^−^ *ompT hsdS gal dcm*	Novagen
ET12567/pUZ8002	*dam-13*::Tn9 *dcm-6 hsdM*; containing the non-transmissible RP4 derivative plasmid pUZ8002	Huang and Grove, 2013
**Plasmids**		
pET-28a (+)	*E. coli* expression vector	Novagen
pLU-02	LmbU_1–161_ cloned in *Nde*I/*Eco*RI sites of pET-28a (+)	This study
pLU-03	LmbU_86–225_ cloned in *Nde*I/*Eco*RI sites of pET-28a (+)	This study
pLU-04	LmbU_1–142_ cloned in *Nde*I/*Eco*RI sites of pET-28a (+)	This study
pLU-05	LmbU_1–131_ cloned in *Nde*I/*Eco*RI sites of pET-28a (+)	This study
pLU-06	LmbU_58–225_ cloned in *Nde*I/*Eco*RI sites of pET-28a (+)	This study
pLU-07	LmbU_58–161_ cloned in *Nde*I/*Eco*RI sites of pET-28a (+)	This study
pLU-13	LmbU_113–225_ cloned in *Nde*I/*Eco*RI sites of pET-28a (+)	This study
pLU-14	LmbU_C12G_ cloned in *Nde*I/*Eco*RI sites of pET-28a (+)	This study
pLU-15	LmbU_C63G_ cloned in *Nde*I/*Eco*RI sites of pET-28a (+)	This study
pLU-16	LmbU_R101A_ cloned in *Nde*I/*Eco*RI sites of pET-28a (+)	This study
pLU-17	LmbU_R102A_ cloned in *Nde*I/*Eco*RI sites of pET-28a (+)	This study
pSET152	Integrative vector based on ΦC31 integrase	Bierman et al., 1992
pATE152	pSET152 carrying *xylTE* reporter gene controlled by *lmbAp* promoter	Hou et al., 2018
pEU139	pIB139 with *lmbU* inserted downstream of *ermE^∗^p*	Hou et al., 2017
pAU01	pATE152 inserted with LmbU expression cassette	This study
pAU02	pATE152 inserted with LmbU_1–161_ expression cassette	This study
pAU03	pATE152 inserted with LmbU_86–225_ expression cassette	This study
pAU06	pATE152 inserted with LmbU_58–225_ expression cassette	This study
pAU07	pATE152 inserted with LmbU_58–161_ expression cassette	This study
pUTE152	pSET152 carrying *xylTE* reporter gene controlled by *lmbUp* promoter	This study

### Construction, Overexpression, and Purification of LmbU and Its Variants in *E. coli*

To construct LmbU truncated variants, DNA fragments covering different regions of *lmbU* gene were amplified by PCR using primer pairs U02-F28a/R28, U03-F28a/R28, U04-F28a/R28, U05-F28a/R28, U06-F28a/R28, U07-F28a/R28, and U13-F28a/R28 listed in Supplementary Table S1. The amplified DNA fragments were inserted into the *Nde*I/*Eco*RI restriction sites of the pET-28a (+) vector, resulting in various expression plasmids pLU-02, pLU-03, pLU-04, pLU-05, pLU-06, pLU-07, and pLU-13, which were used for expression of LmbU_1–161_, LmbU_86–225_, LmbU_1–142_, LmbU_1–131_, LmbU_58–225_, LmbU_58–161_, and LmbU_113–225_.

To construct LmbU point-mutant variants, DNA fragments covering different upstream or downstream within *lmbU* genes were, respectively amplified by PCR using the primer pairs listed in Supplementary Table S1. Among them, primer pairs U-P1/U-RR-P2 with U-RR-P3/U-P4 were used for combined mutation of R101 and R102, primer pairs U-P1/U-R101-P2 with U-R101-P3/U-P4 were used for mutation of R101, primer pairs U-P1/U-R102-P2 with U-R102-P3/U-P4 were used for mutation of R102, primer pairs U-P1/U-C63-P2 with U-C63-P3/U-P4 were used for mutation of C63. The mutations were introduced by primers P2 and P3, R was replaced with A, and C was replaced with G. The corresponding DNA fragments of upstream and downstream of *lmbU* were inserted into the *Nde*I/*Eco*RI restriction sites of the pET-28a (+) vector by using Super Efficiency Fast Seamless Cloning kits (DoGene, China), resulting in various expression plasmids pLU-08, pLU-16, pLU-17, and pLU-15, which were used for expression of LmbU_RR_, LmbU_R101A_, LmbU_R102A_, and LmbU_C63G_. In addition, to construct LmbU point-mutant variant LmbU_C63G_, a DNA fragment was amplified by PCR using primer pairs U-C12-P1/U-R28a, and inserted into the *Nde*I/*Eco*RI restriction sites of the pET-28a (+) vector, resulting in expression plasmids pLU-14.

The obtained plasmids were transformed into *E. coli* BL21 (DE3) for protein expression as described previously (Hou et al., 2017). Briefly, The strains were cultivated at 37°C until OD_600_ reached about 0.6, isopropyl–D-1-thiogalactopyranoside (IPTG) was added and the cultures were then incubated at 16°C overnight. The proteins were released by sonication on ice and were purified using nickel-iminodiacetic acid–agarose chromatography (WeiShiBoHui, China). After dialysis using binding buffer (10 mM Tris–HCl, pH 8.0, 1 mM EDTA, 0.2 mM dithiothreitol, 20 g/ml bovine serum albumin, 1.2% glycerol) and concentration using 10 or 3-kDa-cutoff centrifugal filter units (Millipore, Billerica, MA, United States), the proteins were analyzed and quantified using 12% SDS-PAGE and Bradford assay, respectively (Bradford, 1976).

### Electrophoretic Mobility Shift Assay (EMSA)

Electrophoretic mobility shift assay were carried out as described previously (Hou et al., 2017). Briefly, biotin-labeled probe *lmbAp* (5 ng) was incubated with His_6_-LmbU or variants (different concentrations) in the binding reaction mixture contained 10 mM Tris–HCl (pH 8.0), 1 mM EDTA, 0.2 mM dithiothreitol, 20 g/mL bovine serum albumin, 1.2% glycerol, and 50 g/mL poly (dI-C). After incubation at 28°C for 20 min, the samples were separated on 6% non-denaturing polyacrylamide gels in 0.5 × TBE buffer (54 g/L Tris, 1.86 g/L EDTA and 27.5 g/L boric acid, pH 8.0) in ice-water bath at 100 V, and transferred to the positively charged nylon membrane. The biotin-labeled probes were detected by streptavidin- horseradish-peroxidase (HRP) conjugate and BeyoECL Plus (Beyotime Biotechnology, China). Each experiment was at least repeated two times, and the representative images are shown.

### xylTE Reporter Assays

To analyze the function of LmbU and its variants *in vivo*, we performed *xylTE* reporter assays. DNA fragments covering LmbU expression cassettes (*ermE^∗^p* promoter plus *lmbU* gene or *lmbU* variants) were amplified by PCR using primer pairs E*^∗^*p-lmbU-F/R with pLU-1, pLU-03, and pLU-06 as templates, and using primer pairs E*^∗^*p-lmbU-F/lmbU4-R with pLU-2 and pLU-7 as templates. The amplified fragments were inserted into the *Nhe*I restriction sites of the pATE152 plasmid by using T4 DNA ligase (TAKARA, Japan), resulting in pAU01, pAU02, pAU03, pAU06, and pAU07 plasmids. The obtained plasmids were then introduced into the *lmbU* disruption strain JLUa2 and integrated into the *attB* site of the chromosome to generate reporter strains JAU01, JAU02, JAU03, JAU06, and JAU07. The reporter plasmid pUTE152 was constructed as pATE152 described previously (Hou et al., 2018). The region upstream (relative to the translation start codon) of the *lmbU* gene (-329 *–* 1 bp) was amplified using primer pairs pUxyl-1/pUxyl-2, and the *xylTE* gene was amplified by PCR using primer pair pAxyl-3/pAxyl-4. Two fragments were inserted into the *Pvu*II site of the plasmid pSET152 using Super Efficiency Fast Seamless Cloning kits (Do Gene, China). The obtained plasmid was introduced into wild-type strain NRRL 2936 and *lmbU* disruption strain JLUa2, and integrated into the *attB* site of the chromosome to generate reporter strains LNU and LUU.

The analysis of catechol dioxygenase activity was performed as described previously (Hou et al., 2018). *Streptomyces* strains were grown in YEME medium at 28°C for 1 day, cells were washed in 20 mM potassium phosphate buffer, and suspended in 1 ml sample buffer (100 mM potassium phosphate, pH 7.5, 20 mM EDTA, 10% acetone). Total proteins were harvested by sonication, and quantified using the Bradford method (Bradford, 1976). 20 μl total proteins were added to 180 μl assay buffer (100 mM potassium phosphate, pH 7.5, 1 mM catechol), and were detected at 375 nm at 1, 2, 3, 4, 5, and 6 min, respectively. The activity was calculated as the rate of change per minute per milligram of protein and converted to milliunits per milligram. Data represent means ± standard deviations of results from three independent experiments. Statistical significance is indicated vs. the results of wild-type LmbU by using *T* test (Kim, 2015), ns, not significant; ^∗∗^*P* < 0.01; ^∗∗∗^*P* < 0.001.

### Dimerization Analysis of LmbU

The purified LmbU protein and variants were dealt with different loading buffers, which contained or did not contain SDS or DTT. The total loading buffer consists of 50 mM Tris–HCl (pH 6.8), 2% SDS (m/v), 0.1% bromophenol blue (m/v), 10% glycerin (m/v), and 100 mM DTT. The samples were analyzed by 12% SDS-polyacrylamide gel electrophoresis (SDS-PAGE) (Supplementary Table S2) and stained with Coomassie brilliant blue R-250. The electrophoresis buffer consists of 3 g/L Tris, 19 g/L glycine and 1 g/L SDS. The molecular weights of LmbU dimer (56 kDa) and LmbU monomer (28 kDa) were standardized by the protein marker (TAKARA, Japan).

## Results

### Bioinformatics Analysis of the Structure of LmbU

**FIGURE 1:**
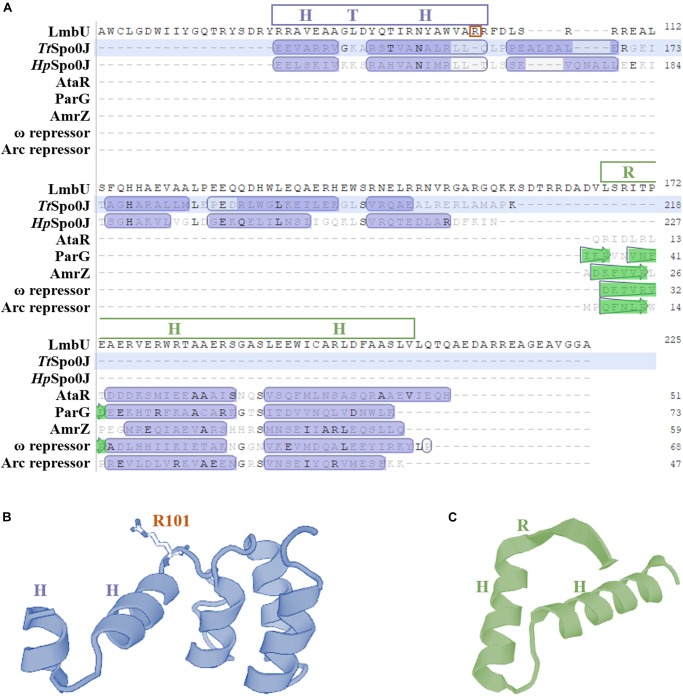
Bioinformatics analysis of the structure of LmbU. **(A)** Structure based alignment of LmbU with partial structural homologs from various bacteria. Putative HTH and RHH motifs are indicated by blue and green boxes, respectively. *Tt*Spo0J is derived from *Thermus thermophiles* (GenBank accession no. WP_011173975.1), *Hp*Spo0J is derived from *Helicobacter pylori* (GenBank accession no. ACJ08256.1), ParG is derived from a multidrug resistance plasmid TP228 from *E. coli* (GenBank accession no. ACV89876.1), AmrZ is derived from *Pseudomonas aeruginosa* (GenBank accession no. APJ53923.1), ω repressor is derived from a plasmid Psm19035 from *Streptococcus pyogenes* (GenBank accession no. AAR27202.1), and Arc repressor is derived from *Salmonella* bacteriophage P22 (GenBank accession no. AAM81381.1). **(B)** Structural modeling of HTH motif. **(C)** Structural modeling of RHH motif. The results were generated by using a online software SwissModel (https://www.swissmodel.expasy.org/interactive).

In our previous study, we have characterized LmbU as a DNA-binding protein involved in lincomycin biosynthesis, and identified the target genes and the binding site of LmbU (Hou et al., 2017). To further investigate the regulatory mechanism of LmbU, we performed bioinformatics analysis of the structure of LmbU. Secondary structure analysis showed that LmbU protein contains 9 α-helices and 2 β-strands. In addition, 14 protein binding regions, one RNA-binding region and 3 DNA-binding regions were predicted in LmbU (Supplementary Figure S1). Structure modeling demonstrated that two potential DBDs, a putative HTH motif including amino acid (aa) 80–102, and a putative ribbon-helix-helix (RHH) motif including aa 167–206, were predicted in LmbU (Figure 1). However, all the templates used for LmbU modeling are not derived from *Streptomyces*, indicating the regulatory pattern of LmbU may be complex and novel compared to other CSRs.

In addition, LmbU homologs naturally exist in hundreds of actinomycetes (Supplementary Figure S2), indicating LmbU homologs might play important roles in metabolism of natural products. Sequence alignment of LmbU with eight selected homologs revealed that the HTH motifs are highly conserved, 11 out of 23 amino acids, especially 10 out of 13 amino acids in the latter helix, are totally identical. In contrast, only 7 out of 40 amino acids in the RHH motif are totally identical (Supplementary Figure S3). These data indicated that the HTH motif might be more important for DNA-binding than the RHH motif.

### Identification of the DNA-Binding Function of HTH and RHH Motifs

**FIGURE 2:**
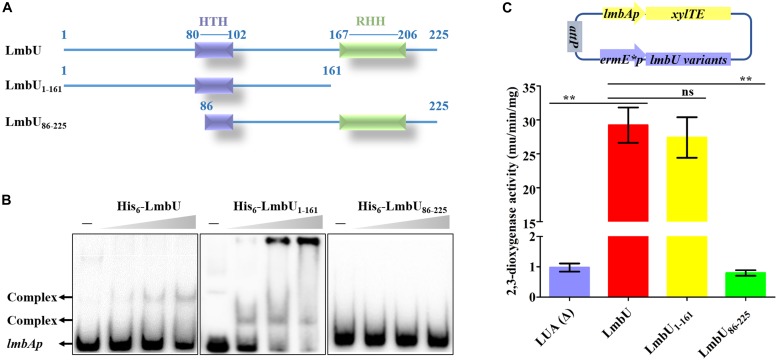
Functional analysis of LmbU variants LmbU_1–161_ and LmbU_86–225_. **(A)** Sketch map of LmbU protein and LmbU variants showing the location of the HTH (aa 80–102) and RHH motif (aa 167–206). **(B)** EMSA analysis of LmbU and variants with *lmbAp* probe. Biotin-labeled *lmbAp* (263 bp, 5 ng) was incubated with increasing concentrations (0, 3.2, 6.4, and 12.8 μM) of His_6_-LmbU, His_6_-LmbU_1–161_, and His_6_-LmbU_86–225_, respectively. The DNA-protein complexes and the free probes are indicated by arrows. **(C)** *XylTE* reporter assay of LmbU and variants to *lmbAp in vivo*. The reporter plasmids were constructed with *xylTE* reporter gene controlled by *lmbAp* and *lmbU* or *lmbU* variants controlled by *ermE^∗^p*. The reporter plasmids were integrated into the *attB* site of the chromosome of JLUa2 to generate reporter strains. In addition, LUA was used as a negative control, which was derived from JLUa2 harboring *xylTE* reporter gene controlled by *lmbAp*. Data represent means ± standard deviations of results from three independent experiments. Statistical significance is indicated vs. the results of wild-type LmbU by using *T* test (Kim, 2015), ns, not significant, ^∗∗^*P* < 0.01.

To identify whether the HTH or/and RHH motifs were relative to the DNA-binding of LmbU, two deletion variants were constructed and expressed, one deleted the HTH motif (designed as LmbU_DH_) and the other deleted the RHH motif (designed as LmbU_DR_) (Supplementary Figure S4A). Unfortunately, His_6_-LmbU_DH_ failed to express in *E. coli* BL21 (DE3). EMSA analysis revealed that His_6_-LmbU_DR_ had the DNA-binding activity (Supplementary Figure S4B), suggesting the RHH motif is not critical for DNA-binding of LmbU. To further verify the DNA-binding activities of RHH and HTH motifs, we expressed and purified the His_6_-LmbU_1–161_ (aa 1–161) and His_6_-LmbU_86–225_ (aa 86_225) variants, which contained the intact HTH motif, and the intact RHH motif, respectively (Figure 2A). EMSA analysis demonstrated that His_6_-LmbU_1–161_ could bind to the *lmbAp* probe, while His_6_-LmbU_86–225_ could not (Figure 2B), which also indicated that the RHH motif is not a critical DBD, and the DBD may exist in LmbU_1–161_. Subsequently, *xylTE* reporter assay was carried out to identify the function of the LmbU variants *in vivo*. The reporter plasmid pATE152, where *xylTE* gene was controlled by *lmbAp* promoter, was introduced into wild-type strain NRRL 2936 and *lmbU* disruption strain JLUa2, resulting in reporter strains LNA and LUA, respectively. The data showed that LmbU activates *lmbAp* promoter (Supplementary Figure S5), which is available and coincident with that of *neo^r^* reporter assay (Hou et al., 2017). In addition, the enzyme activities of total proteins extracted from the cells cultured for 1 day were observed higher than that from the cells cultured for 2 days (Supplementary Figure S5), thus, in the following study, we just detected the enzyme activities at day 1. Reporter plasmids pAUTE1 and pAUTE2 were constructed and introduced into JLUa2 strain, respectively, where the *xylTE* gene was controlled by the *lmbAp* promoter and the *lmbU* mutant genes were controlled by *ermE^∗^p*. The data showed that LmbU_1–161_ rather than LmbU_86–225_ could activate the *lmbAp* promoter (Figure 2C), which is consistent with the results of EMSA, showing that LmbU_1–161_ variant contains a pivotal DBD.

### Verification of the DBD and AID of LmbU

**FIGURE 3:**
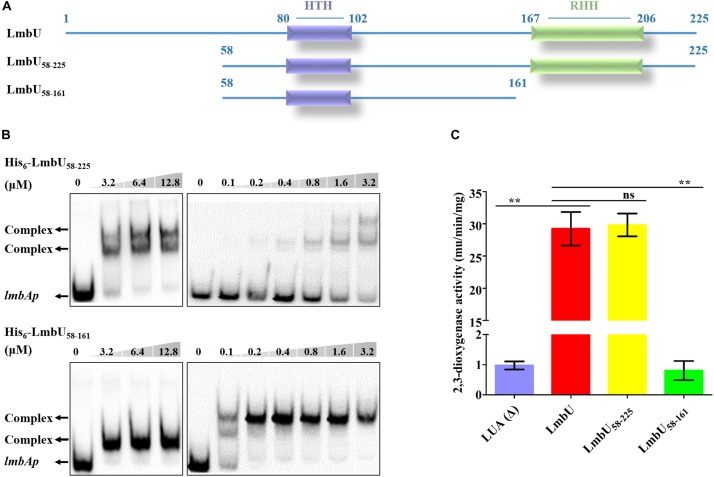
Functional analysis of LmbU variants LmbU_58–225_ and LmbU_58–161_. **(A)** Sketch map of LmbU protein and LmbU variants. LmbU_58–225_ contains intact HTH and RHH motifs, LmbU_58–225_ only contains intact HTH motif. **(B)** EMSA analysis of LmbU and variants with *lmbAp* probe. Biotin-labeled *lmbAp* (263 bp, 5 ng) was incubated with increasing concentrations of His_6_-LmbU_58–225_ and His_6_-LmbU_58–161_, respectively. The DNA-protein complexes and the free probes are indicated by arrows. **(C)** *XylTE* reporter assay of LmbU and variants to *lmbAp in vivo*. Data represent means ± standard deviations of results from three independent experiments. Statistical significance is indicated vs. the results of wild-type LmbU by using *T* test (Kim, 2015), ns, not significant; ^∗∗^*P* < 0.01.

To narrow down the region of potential DBD of LmbU, we further truncated LmbU_1–161_ to the LmbU_1–141_ and LmbU_1–131_ variants. However, both His_6_-LmbU_1–141_ and His_6_-LmbU_1–131_ were failed to express in *E. coli* BL21 (DE3). Therefore, semi-quantitative reverse transcription and polymerase chain reaction (sqRT-PCR) and Western blotting were performed to check the RNA levels and the protein levels of LmbU variants, respectively. The data demonstrated that RNA levels of *lmbU_1_*_*_141_* and *lmbU_1_*_*_131_* had no differences with that of *lmbU*, but protein levels of them were severely reduced compared to that of LmbU (Supplementary Figure S6). Then, we extended LmbU_86–225_ to LmbU_58–225_ (Figure 3A) and performed EMSA. The data revealed that His_6_-LmbU_58–225_ could recover the DNA-binding activity, and the affinity to the target seemed enhanced compared to LmbU. Further EMSA analysis showed that the complex bands were observed when 0.2 μM His_6_-LmbU_58–225_ was added (Figure 3B), but that was observed when 3.2 μM His_6_-LmbU was added, indicating N-terminal of LmbU contains an AID against DNA-binding.

Given that both His_6_-LmbU_1–161_ and His_6_-LmbU_58–225_ have DNA-binding activities, we speculated that the DBD of LmbU was located in the overlapping region of the two variants. Thus, we constructed and expressed the His_6_-LmbU_58–161_ variant (Figure 3A) and performed EMSA. As expected, His_6_-LmbU_58–161_ was found to bind to the *lmbAp* probe as well. In addition, the complex bands were observed when 0.1 μM His_6_-LmbU_58–161_ was added, and 0.2 μM protein could completely impede the migration of the *lmbAp* probe (Figure 3B), indicating that LmbU_58–161_ has a better affinity to the target compared to LmbU and LmbU_58–225_. Thus, we demonstrate that LmbU_58–161_ has a DNA-binding activity, and the HTH motif is located in this region, suggesting the HTH motif is possibly a crucial DBD of LmbU. Furthermore, *xylTE* reporter assays showed that LmbU_58–25_ could activate *lmbAp* promoter, but not like the result of EMSA, the activity of LmbU_58–225_ for *lmbAp* promoter was not enhanced compared to that of LmbU (Figure 3C). While, LmbU_58–161_ could not activate *lmbAp* promoter (Figure 3C), indicating that C-terminal of LmbU performed a certain function to regulate the activity of *lmbAp* promoter *in vivo*.

**FIGURE 4:**
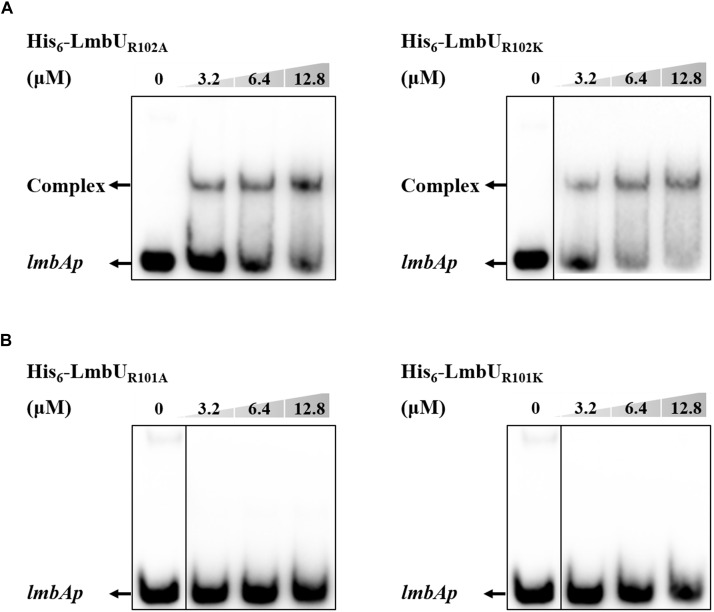
Effect of R102 and R101 on the DNA-binding activity of LmbU. **(A)** EMSA analysis of LmbU variants His_6_-LmbU_R102A_ and His_6_-LmbU_R102K_ with *lmbAp* probe. **(B)** EMSA analysis of LmbU variants His_6_-LmbU_R101A_ and His_6_-LmbU_R101K_ with *lmbAp* probe.

It has been reported that polar and positively charged amino acids are usually important for DNA-binding of regulators, such as arginine (Davis et al., 2013; Bhukya et al., 2014). To further verify whether the HTH motif is responsible for DNA-binding, two arginines in the motif, R101 and R102, were, respectively substituted with either an alanine or a similarly charged lysine, resulting in LmbU_R101A_, LmbU_R102A_, LmbU_R101K_, and LmbU_R102K_. EMSA analysis revealed that His_6_-LmbU_R102A_ and His_6_-LmbU_R102K_ could bind to the *lmbAp* probe (Figure 4A) while His_6_-LmbU_R101A_ and His_6_-LmbU_R101K_ could not (Figure 4B), indicating that the HTH motif is a critical DBD and R101 plays a key role in DNA-binding. These data also demonstrated that the HTH motif, not the RHH motif is the DBD of LmbU.

### Insight Into Regulation of LmbU to *lmbUp*

**FIGURE 5:**
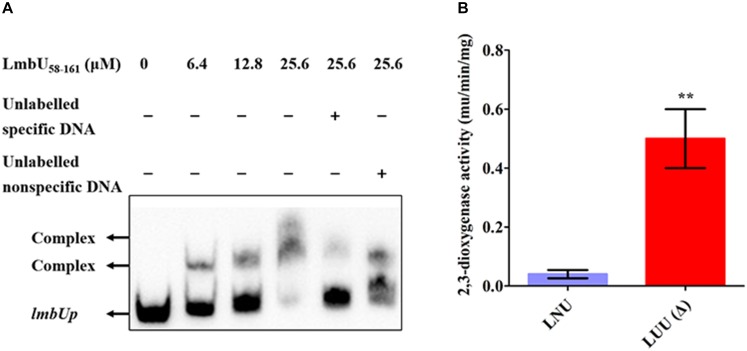
Regulation of LmbU to its own promoter *lmbUp*. **(A)** EMSAs of LmbU 58–161 with *lmbUp* probe. Biotin-labeled *lmbUp* (415 bp, 5 ng) probes were incubated with increasing His6-LmbU 58–161 (0, 6.4, 12.8, and 25.6 μM). EMSAs with 200-fold excess of unlabeled specific DNA or non-specific DNA were added as controls, to confirm specificity of the band shifts. The DNA-protein complexes and the free probes are indicated by arrows. **(B)** *XylTE* reporter analysis of the effect of LmbU to *lmbUp in vivo*. LNU, wild-type strain NRRL 2936 harboring the reporter plasmid pUTE152; LUU, *lmbU* disruption strain JLUa2 harboring the reporter plasmid pUTE152. Data represent means ± standard deviations of results from three independent experiments. Statistical significance is indicated vs. the results of wild-type LmbU by using *T* test (Kim, 2015), ^∗∗^*P* < 0.01.

In our previous study, we found that LmbU regulates the *lmbC*, *lmbK* and *lmbU* genes, but does not bind to their promoters (Hou et al., 2017). Considering the DNA-binding activities of LmbU_58–225_ and LmbU_58–161_ were enhanced compared to that of LmbU, we performed EMSA using LmbU_58–225_ and LmbU_58–161_ with *lmbCp*, *lmbKp* and *lmbUp* probes, the P_V -W_3 probe was used as a positive control. The data showed that both His_6_-LmbU_58–225_ and His_6_-LmbU_58–161_ could not bind to the *lmbCp* and *lmbKp* probes, but seemed to bind to the *lmbUp* probe (Supplementary Figure S7). Subsequently, further EMSA with competition analysis were carried out using His_6_-LmbU_58–225_ and LmbU_58–161_ with the *lmbUp* probe. The results showed that both of the two variants can bind to *lmbUp* directly and specifically with a concentration-dependent manner (Figure 5A and Supplementary Figure S8). In addition, *xylTE* reporter assay showed that LmbU represses the activity of the *lmbUp* promoter *in vivo* (Figure 5B), indicating that LmbU might regulate the activity of *lmbUp* promoter by binding to *lmbUp* in a different pattern compared to *lmbAp* and *lmbWp*.

### Identification of Dimerization of LmbU

**FIGURE 6:**
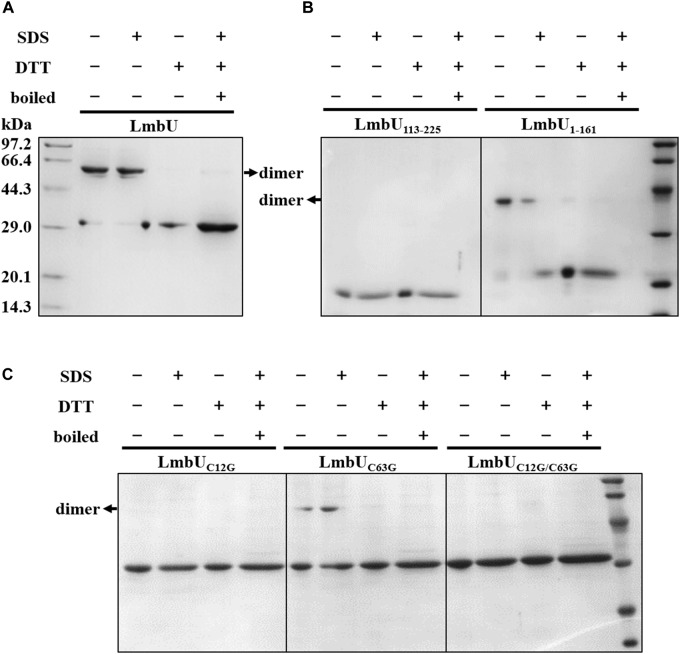
Dimerization analysis of LmbU. **(A)** The LmbU protein was dealt with SDS, DTT, or was boiled for 5 min. −, not dealt with; + dealt with. **(B)** Dimerization analysis of LmbU variants LmbU113-225 and LmbU1-161. **(C)** Effect of C12 and C63 on LmbU dimer.

Generally, regulatory proteins perform their functions by forming homodimers (Bhukya et al., 2014; Hayashi et al., 2014). To investigate the polymeric form of LmbU, we performed SDS-PAGE by using purified LmbU and variants from *E. coli* BL21 (DE3), which were dealt with different loading buffers (containing DTT/SDS or not). The data showed that LmbU could form a homodimer, which was affected by DTT, but not by SDS (Figure 6A), indicating that the homodimer is likely to be formed by disulfide bond among cysteines. Sequence analysis showed that LmbU contains three cysteines, C12, C63, and C196, the first two of which are included in LmbU_1–161_ and the last one is included in LmbU_113–225_. To figure this out, we firstly carried out SDS-PAGE using LmbU_1–161_ and LmbU_113–225_, respectively, and found that the former could form homodimer, but the latter could not (Figure 6B), suggesting the crucial cysteines for dimerization were located in aa 1–161. Then, the two cysteines C12 and C63 were mutated to glycines, resulting in the LmbU_C12G_, LmbU_C63G_ and LmbU_C12G/C63G_ variants. SDS-PAGE analysis revealed that LmbU_C12G_ and LmbU_C12G/C63G_ could not form homodimer, but LmbU_C63G_ could form homodimer partly (Figure 6C), indicating C12 plays a key role in forming LmbU homodimer, and C63 plays a supporting role.

## Discussion

In our previous study, we have demonstrated that a global regulator BldA (Hou et al., 2018) and a CSR LmbU (Hou et al., 2017) are involved in lincomycin biosynthesis. Recently, a TetR-type regulator SLCG_2919 has been proved to negatively regulate lincomycin biosynthesis (Xu et al., 2018). However, the regulatory mechanism of lincomycin biosynthesis is still unclear at present.

Importantly, we found that hundreds of LmbU homologs exist in or outside the BGCs of different natural products derived from a variety of actinomycetes (Supplementary Figure S2), indicating LmbU homologs might play important roles in metabolism and do not only act as CSRs of natural products. Although there are so much LmbU homologs, few studies have been reported. SACE_5599, a homolog of LmbU outside the BGCs of natural products, can regulate not only erythromycin production, but also morphological differentiation in *Saccharopolyspora erythraea* (Kirm et al., 2013), which has been shown to bind to the promoter regions of *lmbAp*, and *lmbWp* within *S. lincolnensis* as well in our previous study (Hou et al., 2017). HmtD, a homolog of LmbU in the BGC of himastatin, positively regulates the biosynthesis of himastatin in *Streptomyces hygroscopicus*, however, the relevant mechanism is still unknown (Xie et al., 2019). In addition, structural prediction of LmbU demonstrated that LmbU protein does not include a known domain similar to that of other CSRs, indicating the regulatory pattern of LmbU and its homologs was novel and complex compared to other CSRs. In the present study, we illuminate the functional domains of LmbU, including DBD and AID, and insight into the regulatory pattern of LmbU.

**FIGURE 7:**
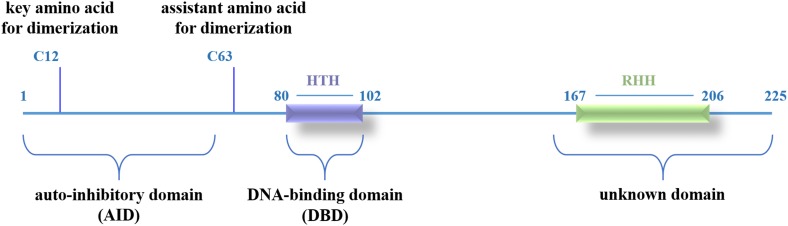
A hypothetical model for the functional domains of LmbU.

We demonstrated that LmbU consists of three functional domains, including a N-terminal AID (aa 1–57), a central DBD (aa 80–102), and a C-terminal unknown domain (aa 162–225) (Figure 7). To our knowledge, HTH motif is the best known and widely used DBD, although LmbU has been shown to bind to the targets by HTH motif as well, the sequence, and structure of HTH motif within LmbU is unlike the most of the regulators in *Streptomyces* (Natsume et al., 2004; Guo et al., 2010; Hayashi et al., 2013), indicating LmbU and its homologs function in a novel regulatory mechanism.

Furthermore, we found that the N-terminal AID represses the DNA-binding of LmbU, and without this domain, LmbU variants can bind to its own promoter region and inhibit transcription of itself, forming negative feedback regulation (Figure 5). Moreover, we found that LmbU homologs within the BGCs of the known antibiotics all contain the N-terminal AID (Supplementary Figure S3), such as HrmB (GenBank Accession No. AEH41782.1) for hormaomycin (Hofer et al., 2011), HmtD (GenBank accession no. CBZ42138.1) for himastatin (Ma et al., 2011), and AcmO (GenBank accession no. ADG27350.1) for actinomycin (Keller et al., 2010). Thus, we speculate that whether LmbU and its homologs within the BGCs of antibiotics function in a more complicated manner, by forming different conformations or different variants. These kinds of cases are not common in the known CSRs, but were found in global regulators. For instance, in *Bacillus subtilis*, the C-terminal of GlnR acts as an auto-inhibitory domain (AID) repressing dimer formation and DNA-binding, when interacting with DNA, GlnR changes its conformational and oligomeric state, resulting in a stable complex (Fisher and Wray, 2008; Wray and Fisher, 2008; Schumacher et al., 2015). In *S. coelicolor*, BldD undergoes degradation of the C-terminal domain, resulting in two forms, which may play roles at vegetative stage or at the late stage of life cycle, respectively (Lee et al., 2007). Interestingly, two PmbA (TldE)-TldD family proteins, LmbIH and LmbQ, are found in the lincomycin BGC. As reported, in *E. coli*, TldD and TldE participate in the cleavage of the modified MccB17 precursor peptide to mature antibiotic by forming heterodimer (Allali et al., 2002; Rodriguez-Sainz et al., 1990; Ghilarov et al., 2017). These studies promote us to speculate that LmbU may undergo accurate post-translational modification by LmbIH and LmbQ in the late growth stage, resulting in functional variant, which binds to its own promoter, and inhibits the biosynthesis of lincomycin. However, further studies are needed to confirm these hypotheses.

In addition, we demonstrated that compared to the functional variant LmbU_58–225_, LmbU_58–161_ can bind to the target DNA *in vitro* with a better affinity, but can not activate transcription of the reporter gene *in vivo*. Considering LmbU_1–161_ without aa 162–225 has a similar DNA-binding affinity to LmbU, thus we thought there is no inhibitory domain within the C-terminal, the better affinity of LmbU_58–161_ might be due to the exposure of the DBD domain. In addition, these data showed that the C-terminal amino acids play important roles as an unknown domain, either in structural stability or in interaction with ligands. However, we also found that LmbU_1–161_ has activity to *lmbAp* promoter both *in vitro* and *in vivo*, indicating the C-terminal domain is not necessary, which appeared to be different from the hypothesis mentioned above. In consideration of the unclear regulatory mechanism of LmbU to the target *lmbCp* and *lmbKp* with no identified binding sites, the function of the C-terminal domain needs to be further illuminated.

Generally, transcriptional regulators bind to the target DNA by forming homodimers. For instance, in *Streptomyces griseus*, a global regulator AdpA either binds to two sites with different lengths in the target DNA (type I or type I’), or binds to a single site in the target DNA with one subunit of the homodimer (type II) (Yamazaki et al., 2004); in *S. coelicolor* A3 (2), a γ-butyrolactone receptor CprB interacts with the target DNA through two individual CprB homodimers (Bhukya et al., 2014). Here, we showed that LmbU can form homodimer mainly via C12. And the DNA-binding mode of LmbU need to be further investigated.

In summary, we have demonstrated the functional domains of LmbU, which is a representative of the LmbU family transcriptional regulators. LmbU consists of an N-terminal AID, a central DBD and a C-terminal unknown domain. In addition, LmbU forms homodimer mainly via the C12 *in vitro*. By applying this knowledge, we speculate that the unusual properties of LmbU will be exploited for future applications in the realization of high-yield of lincomycin, and in the functional research of LmbU family proteins.

## Author Contributions

BH, HZ, and HW designed the experiments. BH, XZ, and YK carried out the experiments. BH, JY, HW, and HZ analyzed the data. BH and HW wrote the manuscript. RW discussed the experimental design and contributed to the manuscript. All authors assisted with critical reading of the manuscript.

## Conflict of Interest Statement

The authors declare that the research was conducted in the absence of any commercial or financial relationships that could be construed as a potential conflict of interest.

## Footnotes

1 https://open.predictprotein.org/

2 https://www.swissmodel.expasy.org/interactive

## References

[B1] Allali N. Afif H. Couturier M. Van Melderen L. (2002). The highly conserved TldD and TldE proteins of *Escherichia coli* are involved in microcin B17 processing and in CcdA degradation. *J. Bacteriol.* 184 3224–3231. 10.1128/jb.184.12.3224-3231.2002 12029038 PMC135094

[B2] Arias P. Fernandez-Moreno M. A. Malpartida F. (1999). Characterization of the pathway-specific positive transcriptional regulator for actinorhodin biosynthesis in *Streptomyces coelicolor* A3(2) as a DNA-binding protein. *J. Bacteriol.* 181 6958–6968. 10559161 10.1128/jb.181.22.6958-6968.1999 PMC94170

[B3] Bhukya H. Bhujbalrao R. Bitra A. Anand R. (2014). Structural and functional basis of transcriptional regulation by TetR family protein CprB from *S. coelicolor* A3(2). *Nucleic Acids Res.* 42 10122–10133. 10.1093/nar/gku587 25092919 PMC4150764

[B4] Bierman M. Logan R. O’Brien K. Seno E. T. Rao R. N. Schoner B. E. (1992). Plasmid cloning vectors for the conjugal transfer of DNA from *Escherichia coli* to *Streptomyces* spp. *Gene* 116 43–49. 10.1016/0378-1119(92)90627-2 1628843

[B5] Bown L. Li Y. Berrue F. Verhoeven J. T. P. Dufour S. C. Bignell D. R. D. (2017). Biosynthesis and evolution of coronafacoyl phytotoxin production in the common scab pathogen *Streptomyces scabies*. *Appl. Environ. Microbiol.* 83 e1169–e1117. 28754703 10.1128/AEM.01169-17 PMC5601335

[B6] Bradford M. M. (1976). A rapid and sensitive method for the quantitation of microgram quantities of protein utilizing the principle of protein-dye binding. *Anal. Biochem.* 72 248–254. 10.1006/abio.1976.9999 942051

[B7] Cundliffe E. (2006). Antibiotic production by actinomycetes: the janus faces of regulation. *J. Ind. Microbiol. Biotechnol.* 33 500–506. 10.1007/s10295-006-0083-6 16463161

[B8] Cuthbertson L. Nodwell J. R. (2013). The TetR family of regulators. *Microbiol. Mol. Biol. Rev.* 77 440–475. 10.1128/MMBR.00018-13 24006471 PMC3811609

[B9] Davis J. R. Brown B. L. Page R. Sello J. K. (2013). Study of PcaV from *Streptomyces coelicolor* yields new insights into ligand-responsive MarR family transcription factors. *Nucleic Acids Res.* 41 3888–3900. 10.1093/nar/gkt009 23396446 PMC3616709

[B10] Fisher S. H. Wray L. V. (2008). *Bacillus subtilis* glutamine synthetase regulates its own synthesis by acting as a chaperone to stabilize GlnR–DNA complexes. *Proc. Natl. Acad. Sci. U.S.A.* 105 1014–1019. 10.1073/pnas.0709949105 18195355 PMC2242682

[B11] Ghilarov D. Serebryakova M. Stevenson C. E. M. Hearnshaw S. J. Volkov D. S. Maxwell A. (2017). The origins of specificity in the microcin-processing protease TldD/E. *Structure* 25 1549–1561. 10.1016/j.str.2017.08.006 28943336 PMC5810440

[B12] Guo J. Zhao J. L. Li L. L. Chen Z. Wen Y. Li J. L. (2010). The pathway-specific regulator AveR from *Streptomyces avermitilis* positively regulates avermectin production while it negatively affects oligomycin biosynthesis. *Mol. Genet. Genomics* 283 123–133. 10.1007/s00438-009-0502-2 20012992

[B13] Hasegawa H. Häse C. C. (2009). TetR-type transcriptional regulator VtpR functions as a global regulator in *Vibrio tubiashii*. *Appl. Environ. Microb.* 75 7602–7609. 10.1128/AEM.01016-09 19837838 PMC2794119

[B14] Hayashi T. Tanaka Y. Sakai N. Okada U. Yao M. Watanabe N. (2013). SCO4008, a putative TetR rranscriptional repressor from *Streptomyces coelicolor* A3(2), regulates rranscription of sco4007 by multidrug recognition. *J. Mol. Biol.* 425 3289–3300. 10.1016/j.jmb.2013.06.013 23831227

[B15] Hayashi T. Tanaka Y. Sakai N. Okada U. Yao M. Watanabe N. (2014). Structural and genomic DNA analysis of the putative TetR transcriptional repressor SCO7518 from *Streptomyces coelicolor* A3(2). *FEBS Lett.* 588 4311–4318. 10.1016/j.febslet.2014.09.037 25305383

[B16] Higo A. Horinouchi S. Ohnishi Y. (2011). Strict regulation of morphological differentiation and secondary metabolism by a positive feedback loop between two global regulators AdpA and BldA in *Streptomyces griseus*. *Mol. Microbiol.* 81 1607–1622. 10.1111/j.1365-2958.2011.07795.x 21883521

[B17] Hofer I. Crusemann M. Radzom M. Geers B. Flachshaar D. Cai X. F. (2011). Insights into the biosynthesis of hormaomycin, an exceptionally complex bacterial signaling metabolite. *Chem. Biol.* 18 381–391. 10.1016/j.chembiol.2010.12.018 21439483

[B18] Hou B. Lin Y. Wu H. Guo M. Petkovic H. Tao L. (2017). The novel transcriptional regulator LmbU promotes lincomycin biosynthesis through regulating expression of its target genes in *Streptomyces lincolnensis*. *J. Bacteriol.* 200 e447–e417. 10.1128/JB.00447-17 29038257 PMC5738732

[B19] Hou B. Tao L. Zhu X. Wu W. Guo M. Ye J. (2018). Global regulator BldA regulates morphological differentiation and lincomycin production in *Streptomyces lincolnensis*. *Appl. Microbiol. Biotechnol.* 102 4101–4115. 10.1007/s00253-018-8900-1 29549449

[B20] Huang H. Grove A. (2013). The transcriptional regulator TamR from *Streptomyces coelicolor* controls a key step in central metabolism during oxidative stress. *Mol. Microbiol.* 87 1151–1166. 10.1111/mmi.12156 23320788

[B21] Iqbal M. Mast Y. Amin R. Hodgson D. A. Consortium S. Wohlleben W. (2012). Extracting regulator activity profiles by integration of de novo motifs and expression data: characterizing key regulators of nutrient depletion responses in *Streptomyces coelicolor*. *Nucleic Acids Res.* 40 5227–5239. 10.1093/nar/gks205 22406834 PMC3384326

[B22] Keller U. Lang M. Crnovcic I. Pfennig F. Schauwecker F. (2010). The actinomycin biosynthetic gene cluster of *Streptomyces chrysomallus*: a genetic hall of mirrors for synthesis of a molecule with mirror symmetry. *J. Bacteriol.* 192 2583–2595. 10.1128/JB.01526-09 20304989 PMC2863554

[B23] Kim T. K. (2015). T test as a parametric statistic. *Korean J. Anesthesiol.* 68 540–546. 10.4097/kjae.2015.68.6.540 26634076 PMC4667138

[B24] Kirm B. Maqdevska V. Tome M. Horvat M. Karnièar K. Petek M. (2013). SACE_5599, a putative regulatory protein, is involved in morphological differentiation and erythromycin production in *Saccharopolyspora erythraea*. *Microb. Cell Fact.* 12:126. 10.1186/1475-2859-12-126 24341557 PMC3878487

[B25] Kisker C. Hinrichs W. Tovar K. Hillen W. Saenger W. (1995). The complex formed between Tet repressor and tetracycline-Mg2+ reveals mechanism of antibiotic resistance. *J. Mol. Biol.* 247 260–280. 10.1006/jmbi.1994.0138 7707374

[B26] Kuscer E. Coates N. Challis I. Gregory M. Wilkinson B. Sheridan R. (2007). Roles of rapH and rapG in positive regulation of rapamycin biosynthesis in *Streptomyces hygroscopicus*. *J. Bacteriol.* 189 4756–4763. 10.1128/jb.00129-07 17468238 PMC1913445

[B27] Lee C. J. Won H. S. Kim J. M. Lee B. J. Kang S. O. (2007). Molecular domain organization of BldD, an essential transcriptional regulator for developmental process of *Streptomyces coelicolor* A3(2). *Proteins* 68 344–352. 10.1002/prot.21338 17427251

[B28] Li Q. L. Wang L. F. Xie Y. Y. Wang S. M. Chen R. X. Hong B. (2013). SsaA, a member of a novel class of transcriptional regulators, controls sansanmycin production in *Streptomyces* sp. strain SS through a feedback mechanism. *J. Bacteriol.* 195 2232–2243. 10.1128/JB.00054-13 23475969 PMC3650532

[B29] Li T. Zhao K. X. Huang V. Li D. F. Jiang C. Y. Zhou N. (2012). The TetR-type transcriptional repressor RolR from *Corynebacterium glutamicum* regulates resorcinol catabolism by binding to a unique operator, rolO. *Appl. Environ. Microb.* 78 6009–6016. 10.1128/AEM.01304-12 22706057 PMC3416628

[B30] Liu G. Chater K. F. Chandra G. Niu G. Tan H. (2013). Molecular regulation of antibiotic biosynthesis in *Streptomyces*. *Microbiol. Mol. Biol. Rev.* 77 112–143. 10.1128/MMBR.00054-12 23471619 PMC3591988

[B31] Ma J. Wang Z. Huang H. Luo M. Zuo D. Wang B. (2011). Biosynthesis of himastatin: assembly line and characterization of three cytochrome P450 enzymes involved in the post-tailoring oxidative steps. *Angew. Chem. Int. Ed. Engl.* 50 7797–7802. 10.1002/anie.201102305 21726028

[B32] Mao X. M. Luo S. Zhou R. C. Wang F. Yu P. Sun N. (2015). Transcriptional regulation of the daptomycin gene cluster in *Streptomyces roseosporus* by an autoregulator. AtrA. *J. Biol. Chem.* 290 7992–8001. 10.1074/jbc.M114.608273 25648897 PMC4367297

[B33] Natsume R. Ohnishi Y. Senda T. Horinouchi S. (2004). Crystal structure of a γ-butyrolactone autoregulator receptor protein in *Streptomyces coelicolor* A3(2). *J. Mol. Biol.* 336 409–419. 10.1016/j.jmb.2003.12.040 14757054

[B34] Ohnishi Y. Yamazaki H. Kato J. Y. Tomono A. Horinouchi S. (2005). AdpA, a central transcriptional regulator in the A-factor regulatory cascade that leads to morphological development and secondary metabolism in *Streptomyces griseus*. *Biosci. Biotechnol. Biochem.* 69 431–439. 10.1271/bbb.69.431 15784968

[B35] O’Rourke S. Wietzorrek A. Fowler K. Corre C. Challis G. L. Chater K. F. (2009). Extracellular signalling, translational control, two repressors and an activator all contribute to the regulation of methylenomycin production in *Streptomyces coelicolor*. *Mol. Microbiol.* 71 763–778. 10.1111/j.1365-2958.2008.06560.x 19054329

[B36] Rodriguez-Sainz M. C. Hernandez-Chico C. Moreno F. (1990). Molecular characterization of pmbA, an *Escherichia coli* chromosomal gene required for the production of the antibiotic peptide MccB17. *Mol. Microbiol.* 4 1921–1932. 10.1111/j.1365-2958.1990.tb02041.x 2082149

[B37] Santamarta I. Lopez-Garcia M. T. Kurt A. Nardiz N. Alvarez-Alvarez R. Perez-Redondo R. (2011). Characterization of DNA-binding sequences for CcaR in the cephamycin-clavulanic acid supercluster of *Streptomyces clavuligerus*. *Mol. Microbiol.* 81 968–981. 10.1111/j.1365-2958.2011.07743.x 21696462

[B38] Schumacher M. A. Chinnam N. B. Cuthbert B. Tonthat N. K. Whitfill T. (2015). Structures of regulatory machinery reveal novel molecular mechanisms controlling *B. subtilis* nitrogen homeostasis. *Genes Dev.* 29 451–464. 10.1101/gad.254714.114 25691471 PMC4335299

[B39] Sheldon P. J. Busarow S. B. Hutchinson C. R. (2002). Mapping the DNA-binding domain and target sequences of the *Streptomyces peucetius* daunorubicin biosynthesis regulatory protein. DnrI. *Mol. Microbiol.* 44 449–460. 10.1046/j.1365-2958.2002.02886.x 11972782

[B40] Si D. Urano N. Shimizu S. Kataoka M. (2012). LplR, a repressor belonging to the TetR family, regulates expression of the L-pantoyl lactone dehydrogenase gene in *Rhodococcus erythropolis*. *Appl. Environ. Microbiol.* 78 7923–7930. 10.1128/AEM.01583-12 22941082 PMC3485960

[B41] Tahlan K. Yu Z. Xu Y. Davidson A. R. Nodwell J. R. (2008). Ligand recognition by ActR, a TetR-like regulator of actinorhodin export. *J. Mol. Biol.* 383 753–761. 10.1016/j.jmb.2008.08.081 18804114

[B42] Takano E. Gramajo H. C. Strauch E. Andres N. White J. Bibb M. J. (1992). Transcriptional regulation of the redD transcriptional activator gene accounts for growth-phase-dependent production of the antibiotic undecylprodigiosin in *Streptomyces coelicolor* A3(2). *Mol. Microbiol.* 6 2797–2804. 10.1111/j.1365-2958.1992.tb01459.x 1435258

[B43] Tanaka A. Takano Y. Ohnishi Y. Horinouchi S. (2007). AfsR recruits RNA polymerase to the afsS promoter: a model for transcriptional activation by SARPs. *J. Mol. Biol.* 369 322–333. 10.1016/j.jmb.2007.02.096 17434533

[B44] Uguru G. C. Stephens K. E. Stead J. A. Towle J. E. Baumberg S. McDowall K. J. (2005). Transcriptional activation of the pathway-specific regulator of the actinorhodin biosynthetic genes in *Streptomyces coelicolor*. *Mol. Microbiol.* 58 131–150. 10.1111/j.1365-2958.2005.04817.x 16164554

[B45] van der Heul H. U. Bilyk B. L. McDowall K. J. Seipke R. F. van Wezel G. P. (2018). Regulation of antibiotic production in actinobacteria: new perspectives from the post-genomic era. *Nat. Prod. Rep.* 35 575–604. 10.1039/c8np00012c 29721572

[B46] Wang L. Tian X. Wang J. Yang H. Fan K. Xu G. (2009). Autoregulation of antibiotic biosynthesis by binding of the end product to an atypical response regulator. *Proc. Natl. Acad. Sci. U.S.A.* 106 8617–8622. 10.1073/pnas.0900592106 19423672 PMC2688989

[B47] Wietzorrek A. Bibb M. (1997). A novel family of proteins that regulates antibiotic production in *streptomycetes* appears to contain an OmpR-like DNA-binding fold. *Mol. Microbiol.* 25 1181–1184. 10.1046/j.1365-2958.1997.5421903.x 9350875

[B48] Wilson D. J. Xue Y. Reynolds K. A. Sherman D. H. (2001). Characterization and analysis of the PikD regulatory factor in the pikromycin biosynthetic pathway of *Streptomyces venezuelae*. *J. Bacteriol.* 183 3468–3475. 10.1128/jb.183.11.3468-3475.2001 11344155 PMC99645

[B49] Wray L. V. Fisher S. H. (2008). *Bacillus subtilis* GlnR contains an autoinhibitory C-terminal domain required for the interaction with glutamine synthetase. *Mol. Microbiol.* 68 277–285. 10.1111/j.1365-2958.2008.06162.x 18331450

[B50] Xie Y. Li Q. Qin X. Ju J. Ma J. (2019). Enhancement of himastatin bioproduction via inactivation of atypical repressors in *Streptomyces hygroscopicus*. *Metab. Eng. Commun.* 8:e00084. 10.1016/j.mec.2018.e00084 30671346 PMC6328088

[B51] Xu Y. Li J. Tang Y. Wang N. Tan G. (2018). Negative Involvement of the TetR-type regulator SLCG_2919 in the regulation of lincomycin biosynthesis in *Streptomyces lincolnensis*. *Appl. Environ. Microbiol.* 85:e02091-18. 10.1128/AEM.02091-18 30341075 PMC6293104

[B52] Yamazaki H. Tomono A. Ohnishi Y. Horinouchi S. (2004). DNA-binding specificity of AdpA, a transcriptional activator in the A-factor regulatory cascade in *Streptomyces griseus*. *Mol. Microbiol.* 53 555–572. 10.1111/j.1365-2958.2004.04153.x 15228534

[B53] Yu Z. Reichheld S. E. Savchenko A. Parkinson J. Davidson A. R. (2010). A comprehensive analysis of structural and sequence conservation in the TetR family transcriptional regulators. *J. Mol. Biol.* 400 847–864. 10.1016/j.jmb.2010.05.062 20595046

[B54] Zhang Y. Pan G. Zou Z. Fan K. Yang K. Tan H. (2013). JadR^∗^-mediated feed-forward regulation of cofactor supply in jadomycin biosynthesis. *Mol. Microbiol.* 90 884–897. 10.1111/mmi.12406 24112541

[B55] Zou Z. Z. Du D. Y. Zhang Y. Y. Zhang J. H. Niu G. Q. Tan H. R. (2014). A γ-butyrolactone-sensing activator/repressor, JadR3, controls a regulatory mini-network for jadomycin biosynthesis. *Mol. Microbiol.* 94 490–505. 10.1111/mmi.12752 25116816

