# Supplementary material for: LmbU, a Cluster-Situated Regulator for Lincomycin, Consists of a DNA-Binding Domain, an Auto-Inhibitory Domain, and Forms Homodimer
Source: Front Microbiol. 2019 May 3;10:989. doi: 10.3389/fmicb.2019.00989 (PMC6510168; doi:10.3389/fmicb.2019.00989)
Supplement: Supplementary file 1 [file Data_Sheet_1.PDF]

Supplemental Material

Characterization of the DNA-binding domain, auto-inhibitory domain and dimerization of LmbU, a cluster-situated regulator for lincomycin biosynthesis

Bingbing Hou[a], Xiaoyu Zhu[a], Yajing Kang[a], Ruida Wang[a], Haizhen Wu[a,b], Jiang Ye[a,]*, Huizhan Zhang[a,b,]*

[a]State Key Laboratory of Bioreactor Engineering, East China University of Science and Technology, Shanghai, China

[b]Department of Applied Biology, East China University of Science and Technology, Shanghai, China

Running head: Characterization of the functional domains of LmbU.

***Address correspondence to:** Jiang Ye; Huizhan Zhang

yyjj413@163.com

huizhzh@ecust.edu.cn

Tel: +86-21-64252515

Fax: +86-21-64252515

Table of Contents:

1. **Supplementary Figure S1.** Secondary structure analysis of LmbU.
2. **Supplementary Figure S2.** Phylogenetic analysis of LmbU and its homologues in a hundred species.
3. **Supplementary Figure S3.** Sequence alignment of LmbU and its homologues derived from various species.
4. **Supplementary Figure S4.** EMSA assays of LmbU variant $LmbU_{DR}$ with *lmbAp* probe.
5. **Supplementary Figure S5.** *XylTE* reporter assay of LmbU to *lmbAp in vivo*.
6. **Supplementary Figure S6.** Expression analysis of $LmbU_{1-131}$ and $LmbU_{1-142}$.
7. **Supplementary Figure S7.** EMSAs assays of LmbU variants $LmbU_{58-225}$ (A) and $LmbU_{58-161}$ (B) with the *lmbCp*, *lmbKp* and *lmbUp* probes.
8. **Supplementary Figure S8.** EMSAs assays of LmbU variants $LmbU_{58-225}$ with the *lmbUp* probes.
9. **Supplementary Table S1.** Primers used in this study.
10. **Supplementary Table S2.** Formula of the 12% SDS-PAGE.
11. **Supplemental Materials and methods**

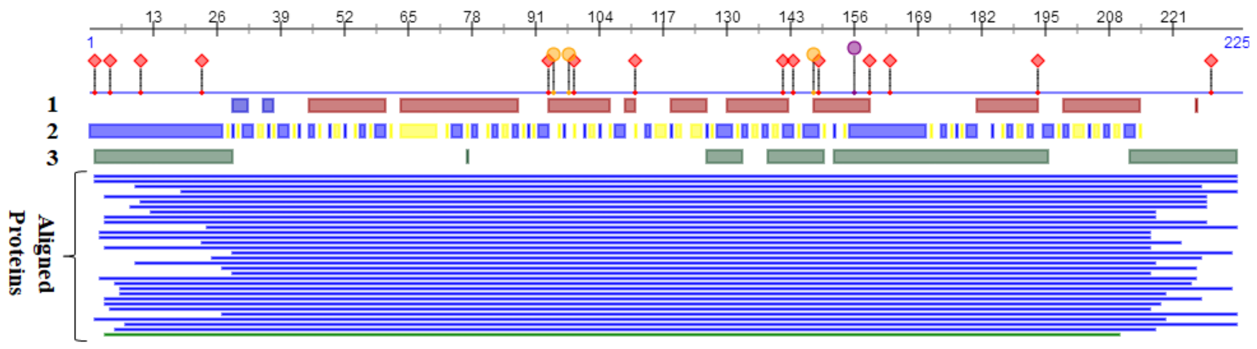

Figure S1. Secondary structure analysis of LmbU. The analysis was carried out by PredictProtein (https://www.predictprotein.org/). The red rhombuses indicate protein binding regions, the yellow circles indicate DNA-binding regions and the purple circle indicates RNA-binding region, that were predicted from two methods, ISIS2 (protein-protein binding sites) and Some NA (polynucleotide binding sites). The red rectangles indicate α-helices, the blue rectangles in line 1 indicate β-strands, the blue rectangles in line 2 indicate exposed regions, the yellow rectangles in line 2 indicate buried regions, and the atrovirens rectangles in line 3 indicate disordered regions. Intrinsically disordered proteins are predicted by Meta-Disorder (MD) from protein sequences. The prediction is based on a system of neural networks that combines the outputs from several original prediction methods (NORSnet, DISOPRED2,PROFbval and Ucon), with the evolutionary profiles and sequence features that correlate with the protein disorder such as predicted solvent accessibility and protein flexibility.

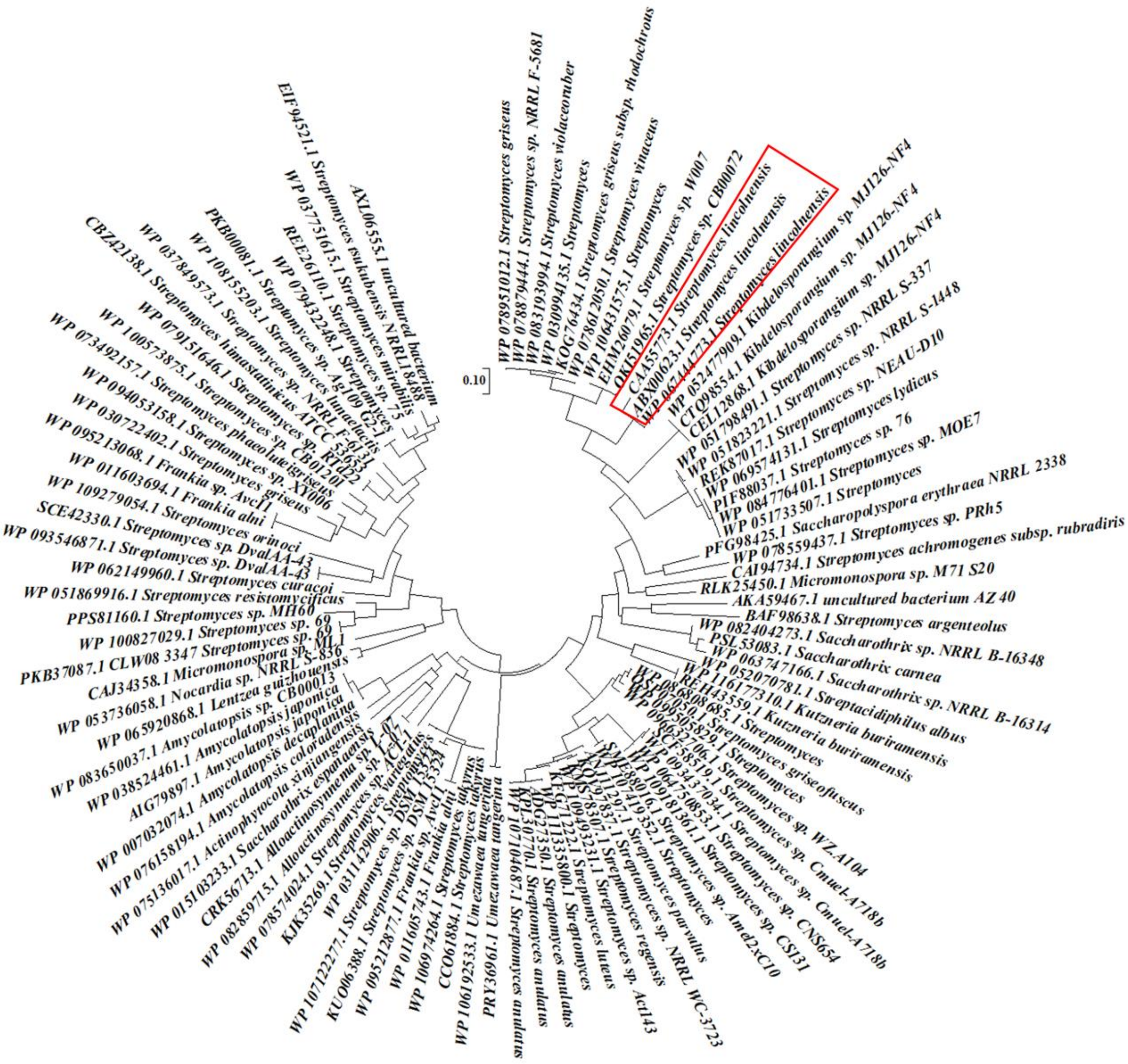

Figure S2. Phylogenetic analysis of LmbU and its homologues in a hundred species. The evolutionary history of them was inferred by MEGA 7.0.14 using the maximum likelihood method (Bown et al., 2017, Hou et al., 2018b). The GenBank accession numbers of LmbU are also labeled in the figure. LmbU derived from *S. lincolnensis* is indicated by red box.

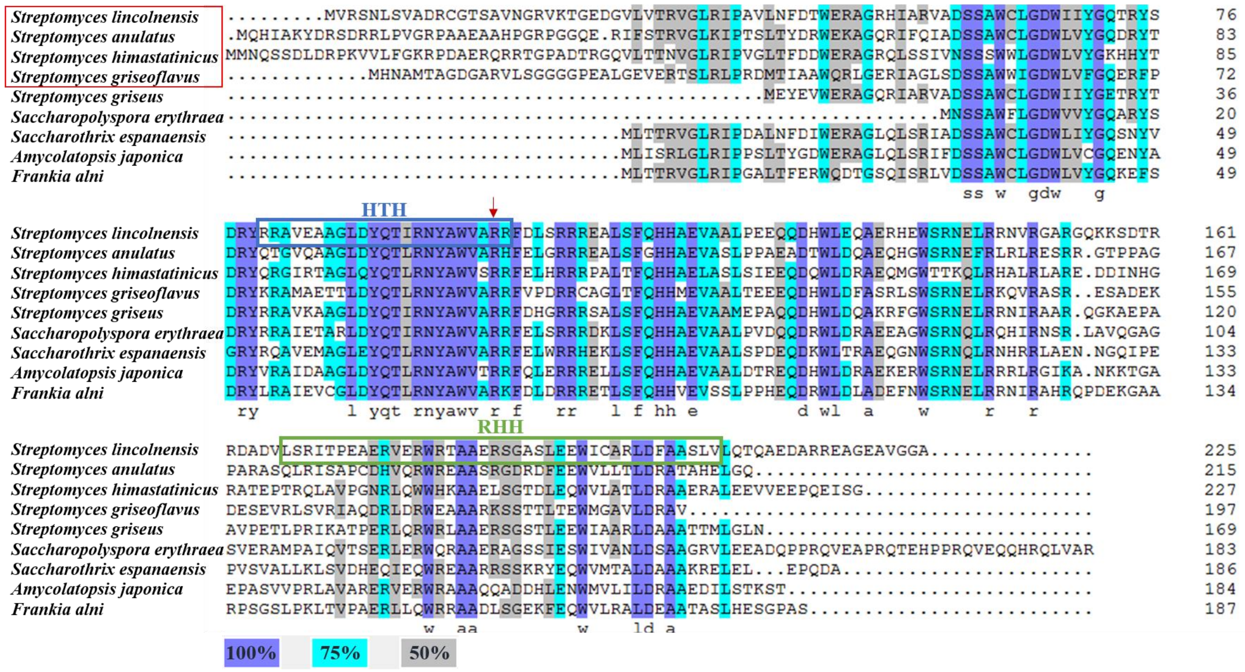

Figure S3. Sequence alignment of LmbU and its homologues derived from various species. HTH motif is indicated by blue box, and RHH motif is indicated by green box. The crucial amino acid R101 is indicated by red arrows. The red box presents LmbU and its homologues within the BGCs of the known antibiotics.

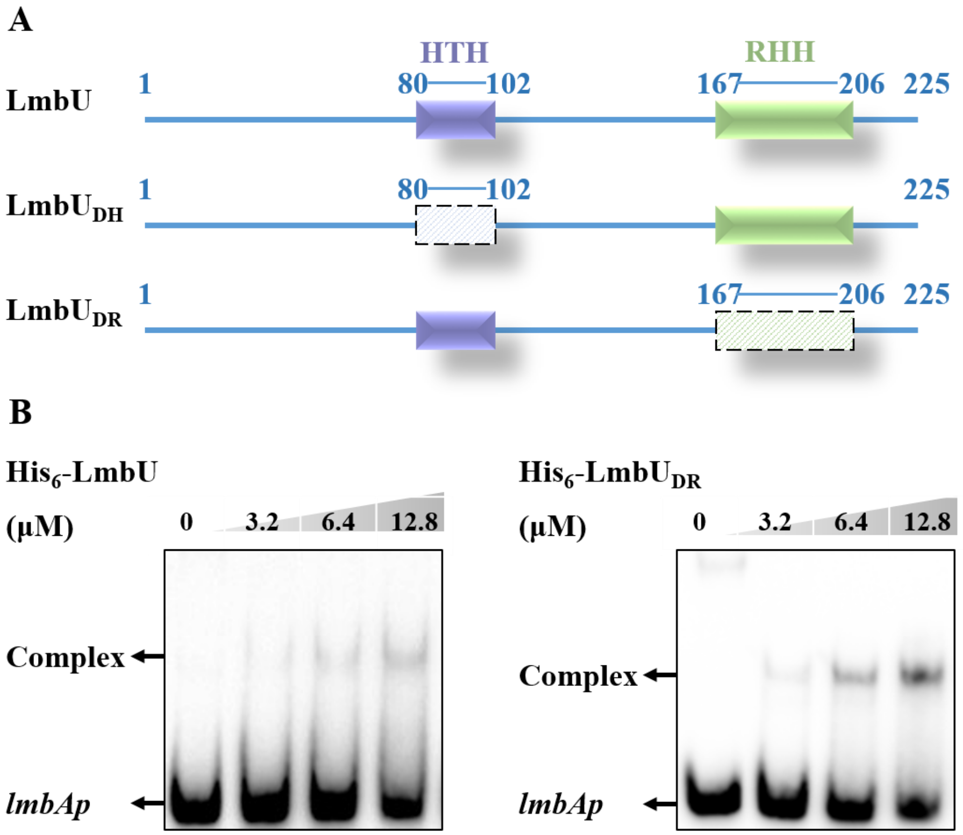

Figure S4. (A) Sketch map of LmbU protein with LmbU variants $LmbU_{DH}$ and $LmbU_{DR}$, which lack of the HTH motif and the RHH motif, respectively. (B) EMSA assays of LmbU variant $LmbU_{DR}$ with *lmbAp* probe. Biotin-labeled *lmbAp* (263 bp, 5ng) was incubated with increasing concentrations (0, 3.2, 6.4 and 12.8 μM) of His_6- $LmbU_{DR}$. The DNA-protein complexes and the free probes are indicated by arrows.

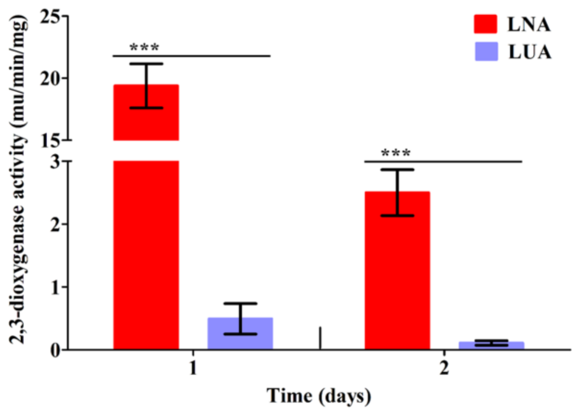

Figure S5. *XylTE* reporter assay of LmbU to *lmbAp in vivo*. The reporter plasmids were constructed with *xylTE* reporter gene controlled by *lmbAp* and *lmbU* controlled by *ermE*p*. The results were achieved from three independent experiments and showed as means ± standard deviations.

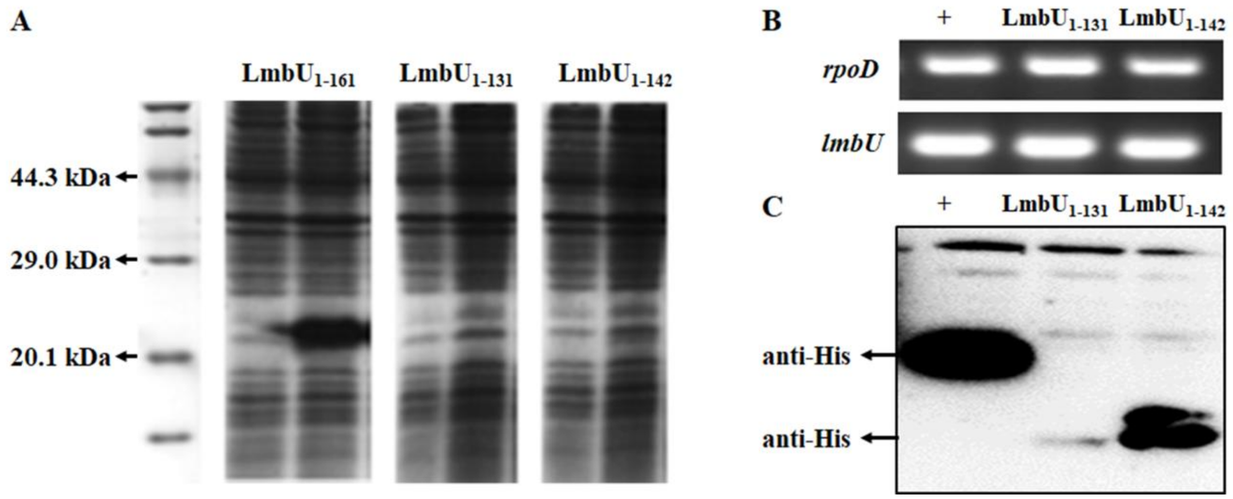

Figure S6. Expression analysis of $LmbU_{1\text{-}131}$ and $LmbU_{1\text{-}142}$. (A) SDS-PAGE of total proteins from *E. coli* BL21 (DE3)/pLU02 ($LmbU_{1\text{-}161}$), *E. coli* BL21 (DE3)/pLU05 ($LmbU_{1\text{-}131}$) and *E. coli* BL21 (DE3)/pLU04 ($LmbU_{1\text{-}142}$). Left lane, IPTG (-); right lane, IPTG (+). (B) SqRT-PCR analysis of transcription of *lmbU* (+) and its mutants. Transcription of *rpoD* was used as control. (C) Western blotting analysis of translation of LmbU (+) and its mutants.

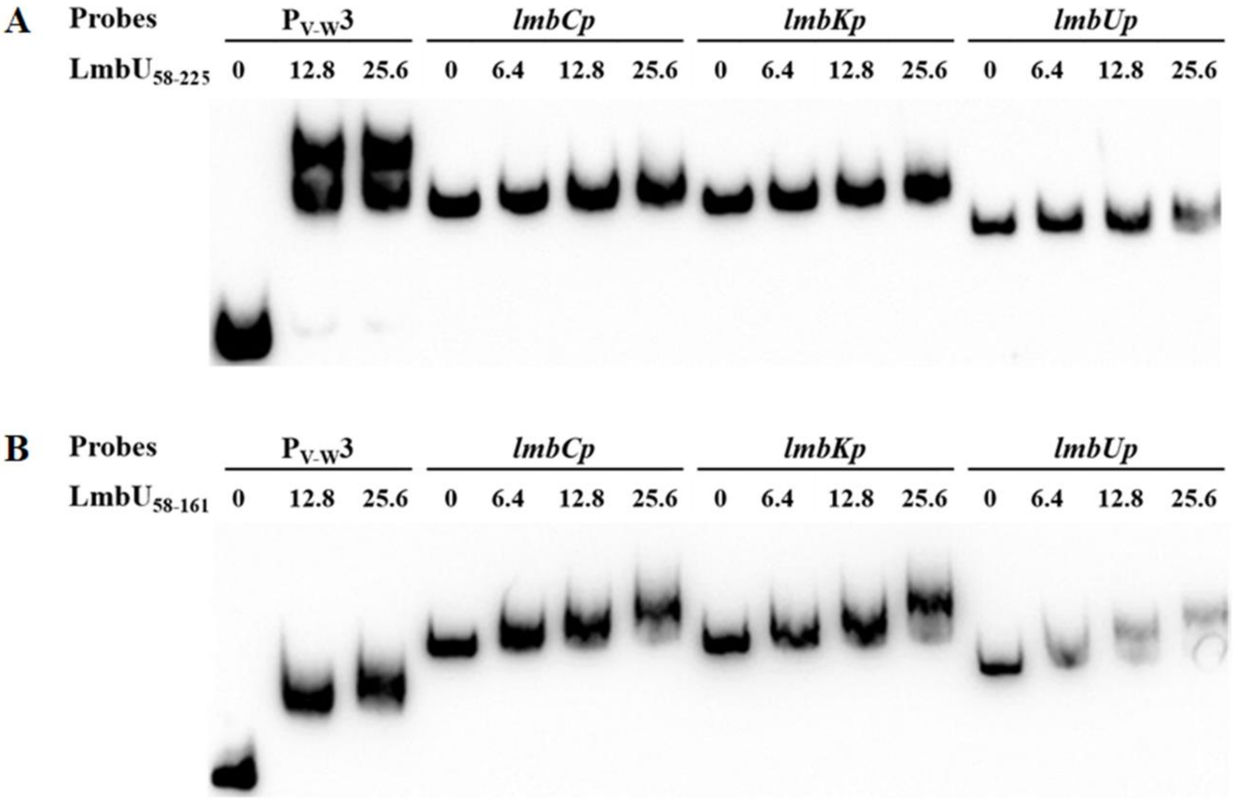

Figure S7. EMSAs assays of LmbU variants $LmbU_{58-225}$ (A) and $LmbU_{58-161}$ (B) with the *lmbCp*, *lmbKp* and *lmbUp* probes. Pv-w3 probe was used as control.

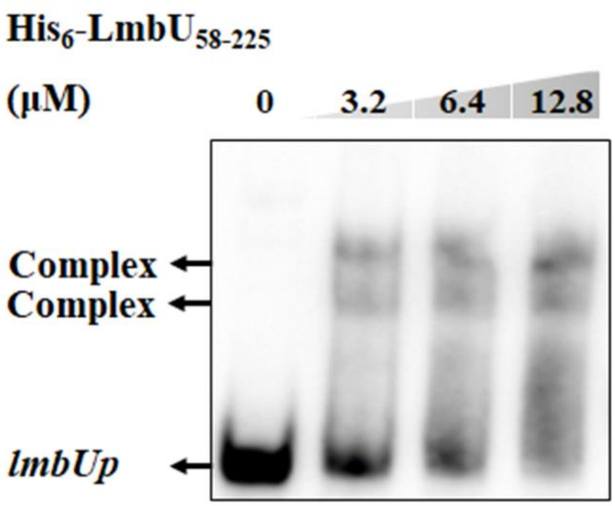

Figure S8. EMSAs assays of LmbU variants $LmbU_{58-225}$ with the *lmbUp* probes.

Table S1. Primers used in this study.

Primer	Sequence(5' to 3')
**Construction of the recombinant proteins**	
U02-F28a	CATTTCACATATGGTGAGGTCGAATTTATCGGTTG
U02-R28a	GCGGAATTCTTAGCGGGTGTCGGACTTCTTCTGC
U03-F28a	CATTTCACATATGGGTCTGGACTACCAGACGATCC
U03-R28a	GCGGAATTCGTGGAGGTGATGGCTGGTGGGC
U04-F28a	CATTTCACATATGGTGAGGTCGAATTTATCGGTTG
U04-R28a	GCGGAATTCTTAGCGGGACCACTCGTGCCTCTC
U05-F28a	CATTTCACATATGGTGAGGTCGAATTTATCGGTTG
U05-R28a	GCGGAATTCTTACCAGTGGTCCTGCTGTTCCTCCG
U06-F28a	CATTTCACATATGGACTCATCGGCCTGGTGTCTGGG
U06-R28a	GCGGAATTCGTGGAGGTGATGGCTGGTGGGC
U07-F28a	CATTTCACATATGGACTCATCGGCCTGGTGTCTGGG
U07-R28a	GCGGAATTCTTAGCGGGTGTCGGACTTCTTCTGC
U-P1	GGTGCCGCGCGGCAGCCATATGGTGAGGTCGAATTTATCGGTTG
U-RR-P2	GAGGTCGAAGGCGGCTGCCACCCAGGCGTAGTTGCGG
U-RR-P3	TGGCAGCCGCCTTCGACCTCTCCCGCAGGCGTG
U-P4	GCAAGCTTGTCGACGGAGCTCGTGGAGGTGATGGCTGGTGGGC
U-R101-P2	AGGTCGAACCGGGCTGCCACCCAGG
U-R101-P3	TACGCCTGGGTGGCAGCCCGGTTCG
U-R102-P2	AGAGGTCGAAGGCCCGTGCCACCCA
U-R102-P3	CTGGGTGGCACGGGCCTTCGACCTC
U13-F28a	CATTTCACATATGTTCCAGCACCACGCCGAGG
U13-R28a	GCGGAATTCGTGGAGGTGATGGCTGGTGGGC
U-C12-P1	ATTTCACATATGGTGGTGAGGTCGAATTTATCGGTTGCGGACAGGGGTGGGAC

	GTCGGCTGTG
U-C63-P2	GTCGCCCAGACCCCAGGCCGATGAGTCG
U-C63-P3	GGCCTGGGGTCTGGGCGACTGGATC
xylTE* reporter assays *in vivo	
pAxyl-1	CTTCGCTATTACGCCAGAGGTAATGCACCGGATATCG
pAxyl-2	CCTCGTAGCCGGCACTGTTCATGCGTCCACCACCATAAC
pUxyl-1	CTTCGCTATTACGCCAGCGTTGGGTTGCCGCTTTGGATGGTC
pUxyl-2	CCTCGTAGCCGGCACTGTTCATGCGGCTGCCATCCCTTTCTCACG
pAxyl-3	ATGAACAGTGCCGGCTACGAGG
pAxyl-4	GGCCGATTCATTAATGCAGTCAGGTCAGCACGGTCATGAATC
E*p-lmbU-F	GTTAGCTAGCTGGGCTGCAGGTCGACTCTAGTATG
E*p-lmbU-R	ATTGGCTAGCTTAGGCGCCCCCGACGGCCTCC
E*p-lmbU4-R	ATTGGCTAGCTTAGCGGGTGTCGGACTTCTTCTGC

Table S2. Formula of the 12% SDS-PAGE

	Separating gel (10 mL)	stacking gel (5 mL)
ddH_2O	3.3 mL	3.4 mL
30% polyacrylamide	4.0 mL	0.83 mL
1.5 M Tris-HCl, pH 8.8	2.5 mL	
1.0 M Tris-HCl, pH 6.8		0.63 mL
10% SDS	0.1 mL	0.05 mL
10% ammonium persulfate	0.1 mL	0.05 mL
Tetramethylethylenediamine	0.004 mL	0.005 mL

Supplemental Materials and methods

RNA extraction and sqRT-PCR

RNA was extracted from the *E. coli* BL21 (DE3) strains used for expressing LmbU and its variants $LmbU_{1-131}$, $LmbU_{1-142}$, by using High Pure RNA Isolation Kit (Roche Diagnostics, Germany) with the manufacturer's instructions. After 1 h of incubation with RNase-free DNase I (TaKaRa, Japan) at 28 ℃, the concentrations and quality of RNA were analyzed using a spectrophotometer (NanoDrop 2000; Thermo Fisher Scientific). The cDNA from 1 μg RNA was synthesized using reverse transcription Moloney murine leukemia virus (M-MLV) kits (TaKaRa, Japan). The sqRT-PCR conditions were performed as follows: 98°C for 3 min; 40 cycles of 95°C for 20 s, 60°C for 20 s, and 72°C for 20 s; and, finally, 72°C for 5 min. To normalize the gene expression, the *rpoD* gene was treated as the positive internal control.

Western blotting analysis

The *E. coli* BL21 (DE3) strains were grown in LB media, and were washed with phosphate-buffered saline. The samples were sonicated and total proteins were quantified using the Bradford method. The proteins were separated by 12% SDS-PAGE, and transferred to the polyvinylidene fluoride (PVDF) membrane. After incubating with TBS buffer (2% pH 7.5 Tris-HCl, 0.8% NaCl) containing 5% skimmed milk for 2-4 h at 25 °C, the membranes were then incubated with anti-His-tag primary antibodies (AOGMA, USA) and HRP-conjugated goat anti-mouse secondary antibodies (AOGMA, USA) successively, and finally detected by BeyoECL Plus (Beyotime Biotechnology, China).
